# Progress on Poxvirus E3 Ubiquitin Ligases and Adaptor Proteins

**DOI:** 10.3389/fimmu.2021.740223

**Published:** 2021-12-09

**Authors:** Haoran Cui, Yaxian Zhang, Leiliang Zhang

**Affiliations:** ^1^ Department of Clinical Laboratory Medicine, The First Affiliated Hospital of Shandong First Medical University and Shandong Provincial Qianfoshan Hospital, Jinan, China; ^2^ Department of Pathogen Biology, School of Basic Medical Sciences, Shandong First Medical University and Shandong Academy of Medical Sciences, Jinan, China; ^3^ Medical Science and Technology Innovation Center, Shandong First Medical University & Shandong Academy of Medical Sciences, Jinan, China

**Keywords:** poxvirus, ubiquitin, E3 ubiquitin ligase, innate immune evasion, MARCH

## Abstract

Poxviruses have evolved a variety of innate immunity evasion mechanisms, some of which involve poxvirus-encoded E3 ubiquitin ligases and adaptor proteins. Based on their functional domains and ubiquitin transfer mechanisms, these poxvirus-encoded E3 ubiquitin ligases and adaptor proteins can be divided into five categories: PRANC, ANK/BC, BBK, P28/RING, and MARCH proteins. Although the substrates of many poxvirus E3 ubiquitin ligases remain to be discovered, most of the identified substrates are components of the innate immune system. In this review, we discuss the current research progress on poxvirus-encoded E3 ubiquitin ligases and adaptor proteins to provide mechanistic insights into the interplay between these viruses and their hosts.

## Introduction

Members of the family *Poxviridae* are double-stranded DNA viruses that replicate in the cytoplasm of the host cell. After infecting the host, poxviruses usually cause local or systemic purulent skin damage. *Poxviridae* is divided into two subfamilies: *Chordopoxvirinae* and *Entomopoxvirinae*. The typical members of the *Chordopoxvirinae* subfamily are variola virus (VARV), cowpox virus (CPXV), monkeypox virus (MPXV), vaccinia virus (VACV), orf virus (ORFV), myxoma virus (MYXV), and ectromelia virus (ECTV) (1).

The host inhibits the replication and spread of the virus through both the innate and adaptive immune systems. Therefore, immune escape mechanisms are particularly important for the survival of the virus (2). Poxviruses have gradually developed a variety of immune escape strategies during their evolution. For instance, poxviruses encode ubiquitination pathway components that modify the host proteins, directly affecting viral recognition, the generation of antiviral signals and inflammation, and the elimination of the virus.

**Figure 1:**
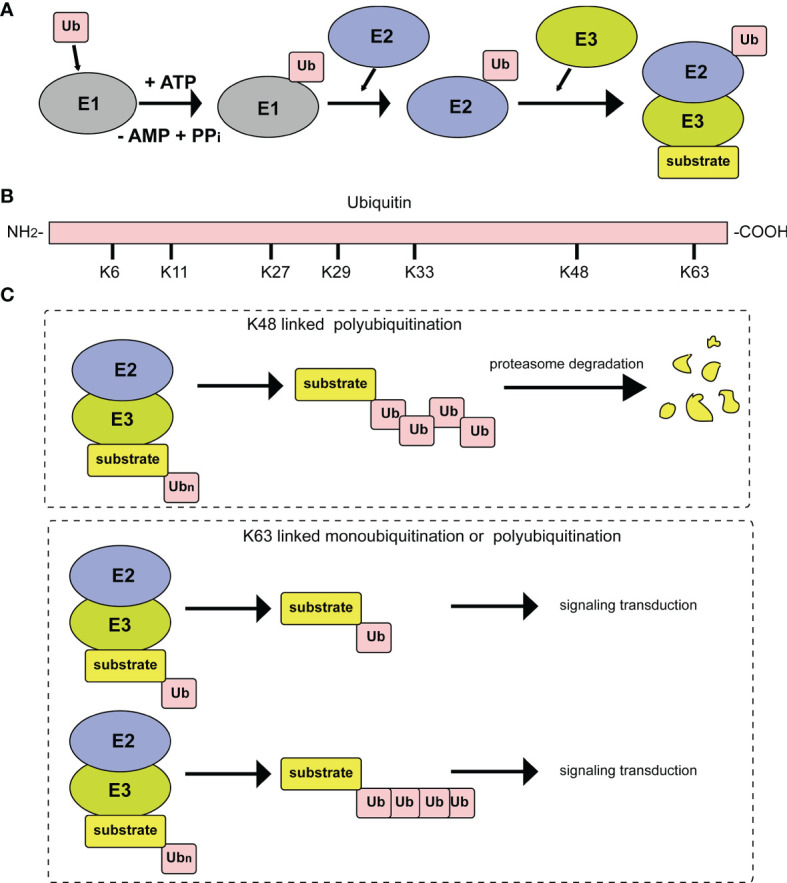
Diagrammatic sketch of the ubiquitin cascade and E3 classification. **(A)** Illustration of the ubiquitin/26S proteome pathway. E1 is activated through ATP hydrolysis and adenosylates the ubiquitin molecule. Then, ubiquitin is successfully transferred to E3 from E1 by the catalysis of E2. With the participation of E3, substrates are modified by the ubiquitin molecule in different linkage modes. **(B)** The seven lysine residues on the ubiquitin molecule that can be ubiquitinated. **(C)** Different ubiquitin molecule linkage types. Upper panel, ubiquitin molecules connect with each other through K48 to form a polyubiquitin chain. Substrates modified by the polyubiquitin chain are degraded by the proteasome pathway. Lower panel, substrate modification by ubiquitin molecules through K63 type monoubiquitylation or polyubiquitylation, which plays important roles in signal transduction.

Ubiquitin consists of 76 amino acids and can be attached to target proteins. The ubiquitination process occurs through an enzymatic cascade. First, ubiquitin needs to be activated by an E1 ubiquitin-activating enzyme; then, the activated ubiquitin is transferred to an E2 ubiquitin-conjugating enzyme; and finally, an E3 ubiquitin ligase transfers ubiquitin to a lysine residue on the target protein (**Figure 1A**) (3). Deubiquitylating enzymes (DUBs) maintain the dynamic state of the cellular ubiquitome by releasing conjugated ubiquitin from proteins and recycling it to maintain the cellular level of free ubiquitin (4). In the above series of enzymatic cascade reactions, the E3 ubiquitin ligase enzyme plays a vital role in the specific recognition of target substrates. At present, 8 types of polyubiquitination modification linkages have been reported. Seven of them involve connections of the glycine at the C-terminus of the ubiquitin molecule to a lysine in the ubiquitin chain, specifically, K6, K11, K27, K29, K33, K48 or K63 (**Figure 1B**). Among those seven polyubiquitination modifications, those at K48 and K63 are the best studied. At least four ubiquitins linked together *via* their Lys48 residues form the ubiquitin chain that triggers degradation by the 26S proteasome (**Figure 1C**). In addition, monoubiquitination or Lys63-linked polyubiquitination functions as a nonproteolytic signal in intracellular trafficking, DNA repair, and signal transduction pathways (**Figure 1C**). The eighth type of ubiquitin linkage is linear ubiquitination, in which the amino group of the methionine residue on ubiquitin is connected to the carboxy group of the glycine residue of another ubiquitin. Ubiquitin-like proteins (UbLs), including NEDD8, SUMO, and ISG15, are biochemically similar to Ub and are also covalently attached to the lysines of their substrates. The conjugation of ubiquitin to UbL, and *vice versa*, can also occur, forming hybrid chains (5).

The E3 ubiquitin ligases in eukaryotes are mainly classified into three types based on their functional domains and ubiquitin transfer mechanisms (6). The most abundant type of ubiquitin ligase is Really Interesting New Gene (RING) E3s. They are characterized by a zinc-binding domain, called a RING domain, or a U-box domain (7). Some RING E3s, such as cullin-RING ligases (CRLs), are composed of multiple subunits. CRLs are composed of a cullin scaffold with a RING-box domain at its N-terminus, an adaptor protein and a substrate receptor. There are several subtypes of CRL ligases. One subtype contains Casitas B-Lineage Lymphoma Proto-Oncogene C (c-Cbl), Mouse Double Minute 2 (Mdm2), and Inhibitor of Apoptosis (IAP) and RING finger proteins (8). Another CRL ligase subtype comprises large protein complexes that include a minimal core element composed of cullin and RING-H2. Another subtype of CRL ligase is the Membrane-Associated RING-CH (MARCH) type. The E3 ligases of the Homologous to the E6AP Carboxyl Terminus (HECT) domain family are another E3 ligase type and are characterized by a conserved HECT domain located at the C-terminus of the protein (9). The other E3 ligase type is RING-between RING-RING (RBR) E3s, named after their two predicted RING domains (RING1 and RING2) and an in-between-RING domain (IBR). In the RBR catalytic process, the RING1 domain recruits ubiquitin-charged E2, and the RING2 domain possesses a catalytic cysteine. In this review, we focus on poxvirus-encoded E3 ubiquitin ligases and adaptor proteins, which are CRL ligases.

## Poxvirus-Encoded E3 Ubiquitin Ligases and Adaptor Proteins

**Figure 2:**
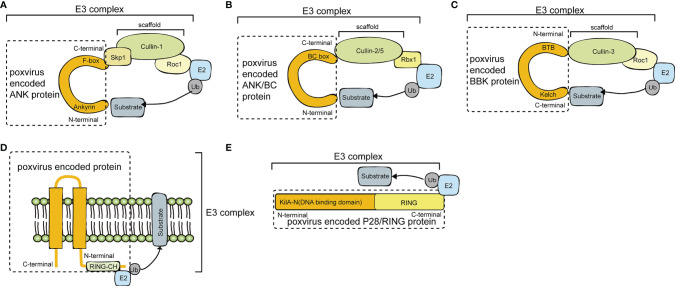
A diagram of poxvirus E3 ubiquitin ligases and adaptor proteins. **(A)** Schematic diagram of Cullin-1 E3 Ub ligases and poxvirus-encoded pox protein repeats of ankyrin-C-terminal domain (PRANC) protein. **(B)** Schematic diagram of Cullin-2/Cullin-5 E3 Ub ligases and poxvirus-encoded ankyrin repeat (ANK)/Elongin B/Elongin C (BC) protein. **(C)** Schematic diagram of Cullin-3 E3 Ub ligases and poxvirus-encoded BTB/Kelch (BBK) protein. **(D)** Schematic diagram of P28/RING protein. **(E)** Schematic diagram of membrane-associated RING-CH (MARCH) protein.

Poxviruses encode many proteins with E3 ubiquitin ligase functions, including pox protein repeats of ankyrin-C-terminal domain (PRANC), ankyrin repeat (ANK)/Elongin B/Elongin C (BC), BTB/Kelch (BBK), P28/RING, and MARCH. Diagrams of the E3 ubiquitin ligases and adaptor proteins encoded by poxvirus are illustrated in **Figure 2** and summarized in **Tables 1**, **2**.

**Table 1:** Summary of poxvirus E3 ubiquitin ligases.

Classification	E3 ubiquitin ligase protein encoded by poxvirus	Substrates
MARCH	M153 (Myxoma virus)	CD4, CD95, MHC-I, FAS (10)
P28/RING	EVM012 (Mouse poxvirus)	not found
	M143 (Myxoma virus)	not found (11)
	P28 (Vaccinia virus)	not found (12)

**Table 2:** Summary of poxvirus adaptor proteins for E3 ubiquitin ligases.

Classification	E3 adaptor protein encoded by poxvirus	Substrates
PRANC	M-T5 (Myxoma virus)	P27/Kip1 (13)
	M148, M149, M150 (Myxoma virus)	not found (14, 15)
	B18R (Vaccinia virus)	not found (16)
	ORFV008, ORFV123, ORFV126 ORFV128, ORFV129 (Orf virus)	not found (17, 18)
	EVM002, EVM005, EVM154 and EVM165 (Ectromelia virus)	IκBα (19, 20)
	CP77 (cowpox virus), C9 (Vaccinia virus)	NF-κB (15)
	viral inducer of RIPK3 degradation (vIRD)	not found (21)
ANK/BC protein	EVM010 (Ectromelia virus)	not found (22)
	MC132 (Molluscum contagiosum virus)	NF-κB p65 (23)
BBK	EVM150, EVM167, EVM18, EVM27 (Ectromelia virus)	not found (24)
	SPPV-019 (Sheeppox virus)	not found (25)
	C2L, F3L, A55R (Vaccinia virus)	not found (26)
	D11L, C18L, G3L, A57R (Vaccinia virus)	not found (27, 28)
	M006, M008, M009, M014, M140 (Myxoma virus)	not found (27, 28)

### PRANC Proteins

Poxviruses usually encode 4 to 5 ANK proteins. The ANK repeat domain, composed of 33 amino acid residues, mainly exists in *Chordopoxvirinae*, often functioning as a protein-protein interaction motif. Approximately 80% of ANK proteins have an F-box domain at the C-terminus, and this F-box domain is shorter than the typical F-box domain in the host cells (17). Specifically, the F-box domain encoded by poxviruses is similar in length to one or two of the three alpha helices of the cellular F-box protein. The M-T5 protein of MYXV; the VACV protein of B18R; the ORFV008, ORFV123, ORFV126, ORFV128, and ORFV129 proteins of ORFV; and EVM002, EVM005, EVM154, and EVM165 of ECTV all contain F-boxes. The Skp, Cullin, F-box (SCF) motif is the recognition motif of E3 ubiquitin ligases that mediate the ubiquitination and degradation of substrates *via* the 26S proteasome (8). In the viral ANK/F-box protein, the F-box motif binds to the N-terminus of the scaffold protein cullin-1 (Cul-1) *via* the adaptor protein S-Phase Kinase Associated Protein 1 (SKP1) (**Figure 2A**). The substrate is then ubiquitinated and degraded by binding to the substrate receptor region of the F-box protein through the degradation sequence. The SCF complex can selectively degrade regulatory proteins, thus modulating a variety of cell activities, such as signal transduction and cell cycle regulation (26).

MYXV encodes M-T5, M148R, M149R and M150, all of which are considered PRANC proteins, as they include N-terminal ANK repeats and a C-terminal F-box structure. The association of M-T5 and Cul-1 reduces P27 expression levels through 26S proteasomal degradation mediated by M-T5/SCF1. The association of M-T5 and Cul-1 maintains the protein level of Akt instead of reducing it through M-T5/SCF1-mediated 26S proteasome degradation. M-T5 can mimic the cellular protein PIKE-A, forming a complex with Akt to induce its activity. M-T5 interacts with Akt to promote the phosphorylation of P27 to regulate apoptosis and cell growth. P27/kip1 belongs to the cell cycle control protein Cip/Kip family and is a negative cell cycle regulator. The continuous phosphorylation and ubiquitination-associated degradation of P27 promote the cell cycle to cross the G0/G1 checkpoint, thereby avoiding programmed cell death due to virus infection (13, 17). The M148 protein encoded by MYXV contains 10 ankyrin repeats and is located in the cytoplasm and nucleus. M149 contains 9 of these ankyrin repeats and is unevenly distributed in the cytoplasm in the form of dots. Neither M148 nor M149 is required for virus replication in tissue culture. However, when MYXV infects rabbits, it acts as a virulence factor (14). The identification of the proteins and target substrates that interact with M148, M149, and M150 requires further research. M150 is another viral protein with an ankyrin repeat sequence, containing nine ankyrin repeats in its N-terminus and an F-box in its C-terminus. This structure is necessary for the pathogenic mechanism of MYXV. The M150 protein localizes to dot-like structures in the nucleus. However, deletion of the eighth ankyrin repeat disrupts its nuclear localization. Therefore, the nuclear localization of M150 depends on the eighth ankyrin repeat. M150 colocalized with p50 of NF-κB in the nucleus in cells stimulated by tumor necrosis factor (TNF). This indicates that M150 may interfere with the NF-κB signaling pathway, but the details of this mechanism remain unclear (14).

The CPXV protein CP77 is a PRANC protein that contains nine ankyrin repeats and a 13 amino acid F-box motif at its C-terminus (15). CP77 blocks TNF-mediated nuclear translocation and activation of the NF-κB subunit p65, while it does not block IκBα phosphorylation. CP77 binds to the NF-κB subunit p65 through six ankyrin repeats in the N-terminus and binds to Cul-1 and SKP1 of the SCF complex through the C-terminal 13 amino acid F-box-like sequence. Through these two pathways, poxvirus CP77 inhibits NF-κB activation and weakens the signal transduction of the natural immune response in cells (15).

In CPXV and other orthopoxviruses, viral inducer of RIPK3 degradation (vIRD) was identified to trigger the ubiquitination and proteasome-mediated degradation of receptor interacting protein kinase 3 (RIPK3) and inhibit necroptosis (21).

ORFV belongs to the genus Parapoxvirus and causes local skin infections in goats, sheep and humans (29). The five proteins ORFV008, ORFV123, ORFV126, ORFV128 and ORFV129 encoded by ORFV all contain F-box domains (18). ORFV008, ORFV123, ORFV126, ORFV128 and ORFV129 interact with the SKP1, Cul-1 and Roc1 proteins of host cells in an F-box-dependent manner, and the interaction of ORFV008 with SCF does not inhibit the E3 ubiquitin ligase function of cellular SCF (17). In summary, poxviruses are likely to use ANK/F-box proteins to recruit target proteins to SCF1 ubiquitin ligase and degrade specific cellular proteins through the ubiquitin protease system of the host to support virus replication.

ECTV causes lethal mousepox in infected mice. The four proteins EVM002, EVM005, EVM154 and EVM165 encoded by ECTV all contain an F-box domain in the C-terminus that interacts with the host SCF complex (30). EVM005 colocalizes with Cul-1 and interacts with Cul-1, SKP1 and Roc1. Deletion of the F-box domain eliminates the interactions among EVM005, Cul-1 and SKP1 (30). One of the key steps in NF-κB activation is the ubiquitination and degradation of IκBα by the cellular SCF β-TrCP-ubiquitin ligase complex. The overexpression of EVM002, EVM005, EVM154 and EVM165 inhibited TNF- or IL-1β-stimulated IκBα degradation and NF-κB subunit p65 nuclear translocation. The inhibition of the NF-κB pathway by EVM005 depends on its F-box domain and the interaction between EVM005 and the SCF complex (19, 20). In A/NCR and C57BL/6 mouse models, virus lacking EVM005 exhibited significantly weakened virulence, indicating that EVM005 is necessary for the toxicity and immune regulation of ECTV (19).

VACV B18R is highly conserved in orthopoxviruses, containing ANK repeats and an F-box domain (16). B18R, together with two other VACV proteins, M2 and C5, has functions in uncoating and in viral DNA replication (31). Yeast two-hybrid screening experiments showed that SKP1A binds to B18R, and this interaction was confirmed by immunoprecipitation experiments, which further showed that the binding of SKP1A to B18R was dependent on the F-box (16).

VACV C9 contains 6 ANK repeats and an F-box domain near the C-terminus, which antagonizes the antiviral state induced by type I interferons (IFNs) in the early stage of VACV replication (32). When VACV infects host cells, pattern recognition receptors (PRRs) bind to pathogen-associated molecular patterns (PAMPs), activating a series of signal cascades to induce the transcription of IFN-encoding genes. Secreted IFNs act on the originating cell or on neighboring cells to activate the JAK-STAT pathway, inducing the transcription of more than 300 interferon-stimulated genes (ISGs). These genes induced by IFNs can protect the host against infection by viruses and other pathogens. Studies have found that C9 interacts with SCF and SCN (COP9 signal body/deubiquitination) complexes. C9 may interact with related proteins through both the F-box and SKP1 to mediate the proteasomal degradation of specific host proteins, such as ISGs. The degradation of ISGs antagonizes the host antiviral status induced by IFNs (32). In addition, the N- and C-terminal portions of C9 bind interferon-induced proteins with tetratricopeptide repeats (IFITs) and ubiquitin regulatory complexes, respectively. Ectopic expression of C9 rescues IFN-induced inhibition of viral DNA replication in IFIT KO cell lines (33).

VACV A49 is phosphorylated at serine 7 but not serine 12, and this phosphorylation is necessary and sufficient for its binding to β-TrCP to antagonize NF-κB (34). A49 inhibits NF-κB activation by molecular mimicry and has a motif near the N-terminus that is conserved in IκBα, β-catenin, HIV Vpu, and some other proteins (34). Phosphorylation of A49 S7 occurs when NF-κB signaling is activated by the addition of IL-1β or overexpression of TRAF6 or IKKβ, the kinase needed for IκBα phosphorylation (34). Thus, A49 is an elegant biological regulation because it becomes an NF-κB antagonist upon activation of NF-κB signaling (34). Interestingly, A49 encodes a second, smaller polypeptide that is expressed *via* leaky scanning translation from methionine 20 and is unable to block NF-κB activation (35). Viruses engineered to express either only the large protein or only the small A49 protein both have lower virulence than wild-type virus and greater virulence than an A49L deletion mutant (35).

In summary, poxviruses encode PRANC proteins that can bind SKP1. The cullin-based E3 ubiquitin ligase consists of three functional components: a catalytic component consisting of a small RING domain protein to recruit the ubiquitin-conjugating enzyme, a cullin scaffold component, and a substrate recognition component that can bind the substrate and recruit it to the vicinity of the catalytic component.

### ANK/BC Proteins

In contrast to ANK repeat/F-box proteins that associate with Cul-1, noncanonical ANK protein 010 encoded by ECTV EVM010 interacts with host cullin-2 (Cul-2) *via* a C-terminal BC box (22). Bioinformatics and mass spectrometry approaches revealed that poxviral ANK ortholog groups IV and VI represent a novel class of viral ANK proteins targeting host Cul-2. In ECTV, ANK/BC proteins suppress the production of CXCL10, CCL5, and IFN, thus inhibiting innate immune signaling (22). Thus, it is speculated that the potential substrate of EVM010 is an innate immune signaling component.

MCTV MC132 was identified to associate with NF-κB subunit p65 by unbiased affinity purification. MC132 targeted p65 for ubiquitin-dependent proteasomal degradation by recruiting p65 to a host Cullin-5 (Cul-5)/BC complex, leading to effective suppression of NF-κB activity and thus benefiting virus replication (23). Poxvirus ANK/BC E3 ligase is illustrated in **Figure 2B**.

### BBK Proteins

The molecular composition and function of the Cul-3-dependent E3 ligase complex has been revealed. In this complex, the binding of BTB domain-containing proteins to their substrates is mediated by Cul-3. Poxvirus is the only virus family known to encode BBK proteins, indicating that poxvirus may interact with Cul-3 to regulate the host ubiquitination pathway.

The BTB domains of the ECTV virus BBK proteins EVM150 and EVM167 bind to the N-terminal region of Cul-3 (**Figure 2C**). Moreover, EVM150 and EVM167 bind to E2-bound ubiquitin and Rocl, and the RING finger protein has the effect of an E3 ubiquitin ligase. In summary, EVM150 and EVM167 rely on Cul-3 to activate their ubiquitin ligase functions, recruiting as-yet unknown substrates for ubiquitination (24). In addition, the BBK proteins EVM18 and EVM27 also interact with Cul-3. It is not clear whether all BBK proteins in poxviruses are related to the ubiquitination pathway.

Studies have found that knocking out the BBK gene in VACV leads to a decrease in the number of mature progeny viruses (36). VACV bearing a BBK gene knockout mutation showed reduced toxicity in the nasal cavities of infected mice. VACV with BBK knockout also exhibited reduced cytopathic changes and formed fewer cell protuberances *in vitro*. The sheeppox virus BBK protein (SPPV)-019 is an important virulence factor. Calcium-independent cell adhesion is reduced in sheep infected with SPPV-019 knockout virus, indicating that SPPV-019 may regulate cell adhesion (25). When injected into sheep through the nasal cavity or an intradermal route, the SPPV-019-knockout virus was very weak, indicating that the SPPV-019 protein plays an important role in the life cycle of the poxvirus (25).

VACV encodes three BBK family proteins: C2L, F3L and A55R (26). The VACV production in mice subcutaneously injected with BBK-knockout VACV was very weak. However, the role of BBK in the ubiquitination pathway of host cells is still unclear. Previous studies showed that the N-terminal BTB-BACK (BB) domain of A55 binds directly to the Cul3 N-terminal domain (Cul3-NTD) (37). According to sequence analysis, D11L, C18L, G3L, and A57R encoded by VACV and M006, M008, M009, M014, and M140 encoded by MYXV also belong to the BBK family of proteins (27, 28).

### MARCH Proteins

MYXV causes widespread lethal multiple myxomatosis in European rabbits (38). The M153R gene of MYXV encodes the E3 ubiquitin ligase M153, which contains a RING-CH domain and two transmembrane domains in its N-terminus (**Figure 2D**). Proteins with such features are referred to as MARCH proteins. In the early stage of MYXV infection, M153 is translocated from the cytoplasm to the endoplasmic reticulum, which in turn upregulates the plasma membrane levels of MHC-I, CD4, activated leukocyte cell adhesion molecule (ALCAM) and the proapoptotic factor Fas/CD95. Those ubiquitinated substrates are then degraded by the lysosomal pathway. M153 inhibits the recognition of MHC-I and death signals by CD8 T lymphocytes and inhibits the recognition of MHC-II molecules by CD4 T lymphocytes (10). During MYXV infection, the RING-CH domain of M153 acts as a ubiquitin ligase to recognize and ubiquitinate the lysine residue in the cytoplasmic tail of CD4, which is responsible for the recognition of MHC-II molecules. Therefore, CD4 ubiquitination inhibits the recognition of MHC-II molecules. The ubiquitination and degradation of immune molecules on the cell surface induced by M153 is an important mechanism by which MYXV suppresses the immune response (10).

### P28/RING Proteins

ECTV causes fatal skin damage in mice, and the P28 protein plays a key role in this process (39). P28 is a virulence factor with E3 ubiquitin ligase activity. P28 contains two functional domains, the DNA binding domain in the N-terminus and the RING domain in the C-terminus. The DNA binding domain is also called the KilA-N domain. The KilA-N domain plays an important role in the cytoplasmic localization of P28 (**Figure 2E**). Fowlpox virus (FWPV) encodes 2 functional P28 ubiquitin ligases, FWPV150 and FWPV157 (40). P28 completely loses its E3 ubiquitin ligase function when the RING domain of P28 is mutated. Therefore, the RING domain plays a role in maintaining its E3 ligase activity. Both the KilA-N domain and RING domain are crucial for the function of P28 ubiquitin ligases. ECTV and VARV P28 interact with the E2 ubiquitin-conjugating enzymes Ubc4 and UbcH5c to degrade substrates. When Ubc13/Uev1A is present, P28 can catalyze the Lys63 ubiquitination of multiple protein substrates (41).

Homologs of P28-containing proteins have also been found in other orthopoxviruses, such as VARV, CPXV, MPXV, VACV, fibroma virus (SFV) and MYXV. P28 in ECTV, M143R in MYXV (11), P28 in VACV (11) and other P28 E3 ubiquitin ligases all contain a RING finger structure. The structure of the P28/RING protein is relatively conserved among different poxviruses. Although the P28/RING protein is not essential for virus replication in cell culture, it is important for the pathogenic mechanism in virus-infected mice (12).

## Conclusion and Perspective

During coevolution with their hosts, poxviruses have incorporated host cell genes into their genomes and adapted them to promote the viral life cycle. In recent years, with in-depth research on the mechanisms of host–virus interactions, research on the function of the E3 ubiquitin ligases encoded by poxviruses has made significant progress. Recently, many new proteins and protein substrates have been discovered using different approaches. For example, bioinformatics analysis and quantitative proteomics were applied to discover that ECTV protein 010 is a noncanonical ANK protein that binds to Cul-2 (22). In contrast, a small interfering RNA (siRNA) approach was used in the discovery of RIPK3 (21). In an extensive crystal structure study, the Cul2-Rbx1-EloBC-VHL complex was revealed, extending the classification of viruses encoding RING E3s (42). Many poxvirus ubiquitin E3 ligases and adapter proteins play important roles in the viral life cycle, including in replication and productive infection. The main functions of poxvirus-encoded E3 ligases are related to host immune evasion (**Tables 1**, **2**).

However, the current understanding of poxvirus E3 ubiquitin ligases is limited. More E3 ligases encoded by poxviruses will be discovered, and many poxvirus E3 ubiquitin ligase substrates remain to be explored. The homologies between genes from different poxviruses and between poxvirus genes and human genes have aided the rational discovery of new poxvirus-encoded E3 ligases, which will facilitate future studies in this field.

The known poxvirus E3 ubiquitin ligases involved in host immune invasion could be developed as potential inhibitors of the host immune system, providing new antiviral strategies. In view of the complex interactions between poxviruses and their hosts, the interplay between poxviruses and the ubiquitin system needs further exploration in the future. The identification of natural substrates of poxvirus E3 ubiquitin ligases will facilitate our understanding of host–virus interactions, and particularly the role of poxvirus E3 ubiquitin ligases in virus infection.

## Author Contributions

LZ conceived the work. HC and YZ draft the manuscript. LZ modified the manuscript. All authors contributed to the article and approved the submitted version.

## Funding

This work was supported by grants from National Natural Science Foundation of China [81871663 and 82072270], Academic promotion programme of Shandong First Medical University [2019LJ001], and Natural Science Foundation of Shandong Province [ZR2021QC095].

## Conflict of Interest

The authors declare that the research was conducted in the absence of any commercial or financial relationships that could be construed as a potential conflict of interest.

## Publisher’s Note

All claims expressed in this article are solely those of the authors and do not necessarily represent those of their affiliated organizations, or those of the publisher, the editors and the reviewers. Any product that may be evaluated in this article, or claim that may be made by its manufacturer, is not guaranteed or endorsed by the publisher.
